# Serum estradiol levels in controlled ovarian stimulation directly affect the endometrium

**DOI:** 10.1530/JME-17-0036

**Published:** 2017-05-24

**Authors:** Kamran Ullah, Tanzil Ur Rahman, Hai-Tao Pan, Meng-Xi Guo, Xin-Yan Dong, Juan Liu, Lu-Yang Jin, Yi Cheng, Zhang-Hong Ke, Jun Ren, Xian-Hua Lin, Xiao-Xiao Qiu, Ting-Ting Wang, He-Feng Huang, Jian-Zhong Sheng

**Affiliations:** 1 The Key Laboratory of Reproductive Genetics (Zhejiang University)Ministry of Education, Hangzhou, Zhejiang, China; 2 Department of Pathology and PathophysiologySchool of Medicine, Zhejiang University, Hangzhou, Zhejiang, China; 3 Shaoxing Women and Children’s HospitalShaoxing, Zhejiang, China; 4 The International Peace Maternity and Child Health HospitalSchool of Medicine, Shanghai Jiao Tong University, Shanghai, China; 5 Department of PathophysiologyWenzhou Medical University, Wenzhou, Zhejiang, China

**Keywords:** IVF-ET, estradiol, endometrium, embryo implantation, proteomics

## Abstract

Previous studies have shown that increasing estradiol concentrations had a toxic effect on the embryo and were deleterious to embryo adhesion. In this study, we evaluated the physiological impact of estradiol concentrations on endometrial cells to reveal that serum estradiol levels probably targeted the endometrium in controlled ovarian hyperstimulation (COH) protocols. An attachment model of human choriocarcinoma (JAr) cell spheroids to receptive-phase endometrial epithelial cells and Ishikawa cells treated with different estradiol (10^−9^ M or 10^−7^ M) concentrations was developed. Differentially expressed protein profiling of the Ishikawa cells was performed by proteomic analysis. Estradiol at 10^−7^ M demonstrated a high attachment rate of JAr spheroids to the endometrial cell monolayers. Using iTRAQ coupled with LC–MS/MS, we identified 45 differentially expressed proteins containing 43 significantly upregulated and 2 downregulated proteins in Ishikawa cells treated with 10^−7^ M estradiol. Differential expression of C_3_, plasminogen and kininogen-1 by Western blot confirmed the proteomic results. C_3_, plasminogen and kininogen-1 localization in human receptive endometrial luminal epithelium highlighted the key proteins as possible targets for endometrial receptivity and interception. Ingenuity pathway analysis of differentially expressed proteins exhibited a variety of signaling pathways, including LXR/RXR activation pathway and acute-phase response signaling and upstream regulators (TNF, IL6, Hmgn3 and miR-140-3p) associated with endometrial receptivity. The observed estrogenic effect on differential proteome dynamics in Ishikawa cells indicates that the human endometrium is the probable target for serum estradiol levels in COH cycles. The findings are also important for future functional studies with the identified proteins that may influence embryo implantation.

## Introduction

Controlled ovarian hyperstimulation (COH) is usually examined by serum estradiol (E_2_) levels for IVF cycles. Serum estradiol (E_2_), up to a certain level, influences embryonic implantation during COH in a concentration-dependent manner (Joo *et al*. 2010). Multiple ovarian follicle maturation will produce supraphysiological serum E_2_ levels that may induce morphologic (Kolb *et al*. 1997) and biochemical (Simón *et al*. 1996) endometrial alterations related to uterine receptivity. In humans (Paulson *et al*. 1990, Ng *et al*. 2000) and mice (Fossum *et al*. 1989), COH led to an increase in estrogen to supraphysiological levels that might compromise embryo implantation, although this contrasts with other reports where elevated E_2_ levels were found to be associated with no change or increased implantation rate (Chenette *et al*. 1990, Sharara & McClamrock 1999). Mirkin *et al*. (2004) reported that high E_2_ levels might impair endometrial receptivity, particularly when COH was used in conjunction with fresh embryo transfers. Clinical studies demonstrated different results. In high responders, regardless of the serum progesterone (P_4_) levels, high serum E_2_ levels on the day of hCG administration were detrimental to uterine receptivity without affecting the embryo quality (Simón *et al*. 1998), while, decreasing E_2_ levels during the preimplantation period by a step-down protocol increased embryo implantation and pregnancy rates (Valbuena *et al*. 1999). On the other hand, one study found high pregnancy rates in higher responder patients (Papageorgiou *et al*. 2002). *In vitro* studies showed that increasing estradiol concentrations from 10^−8^ M to 10^−4^ M are deleterious to embryo adhesion because they directly affected the embryo (Valbuena *et al*. 2001). Recently, a novel mechanism of supraphysiological level of E_2_-induced Aqp5/8-dependent excessive intrauterine fluid accumulation predicted human implantation failure (Zhang *et al*. 2015). Proteomic analysis of the human receptive vs non-receptive endometrium showed differential proteomic repertoire during the window of implantation (Dominguez *et al*. 2009). However, the impact of high E_2_ levels to predict IVF outcome has been the subject of debate.

Following the reported research that serum E_2_ levels impact the pregnancy outcome of *in vitro* fertilization in a concentration-dependent manner, we undertook a more robust and accurate method of protein expression, quantification by mass spectrometry using iTRAQ isobaric tags coupled with 2D nano LC–MS/MS, to screen the comparative proteomic profiling of Ishikawa cells pretreated with different E_2_ (10^−9^ M or 10^−7^ M) concentrations. The Ishikawa cells have the characteristics of glandular and luminal epithelium along with structural proteins and apical adhesiveness to JAr cells and may serve as an excellent model for *in vitro* study of endocrine signaling in the endometrium (Castelbaum *et al*. 1997, Heneweer *et al*. 2005, Mo *et al*. 2006). The Ishikawa cell line also expresses MUC1 markers and steroid (estrogen, progesterone and androgen) receptors and is considered most useful for examining the early events and functional interactions that occur between the luminal epithelium and the trophectoderm (Hannan *et al*. 2010). The expression of proteins was also validated by Western blot. Protein localization in the endometrial luminal epithelium during the receptive phase (LH + 7) was performed by immunofluorescence. The endometrium is receptive to embryonic implantation for a 2-day period (from LH + 7 to LH + 9) corresponding to days 21–23 of the menstrual cycle, in the mid-secretory phase, the so-called ‘window of implantation’ (Dominguez *et al*. 2009).

The proteomic results, combined with immunofluorescent localization, will not only examine the physiological interactions of E_2_ with endometrium in COH cycles that is useful for *in vitro* fertilization (IVF), but should gain new insight into this complex processes as well. Identification of novel biomarkers affected with E_2_ concentrations represents a relatively unexplored area related to embryo implantation. Ingenuity pathway analysis (IPA) was used to determine affected pathways and predicted upstream regulators for the cells as well.

## Materials and methods

### Patients and sample collection

Ethical approval for this study was granted by the Ethics Committee of School of Medicine, Zhejiang University. A written informed consent was obtained from each subject before tissue collection. These women were healthy and not taking any drugs in the past six months. They attended Women’s Hospital, School of Medicine, Zhejiang University to undergo *in vitro* fertilization and embryo transfer (IVF-ET) treatment because of infertility due to tubule pathology without hydrosalpinges. The receptive-phase (LH + 7) endometrial samples were obtained with a biopsy catheter during the spontaneous menstrual cycle for diagnostic purposes before IVF-ET cycle. Shortly after collection, some endometrial tissues were fixed in 10% formalin and processed for paraffin embedding, and others were placed into the DMEM/F-12 medium (HyClone, Logan, UT, USA) for cell culture within 2 h.

### Cells and cell culture

Endometrial tissues were cut into 2–3 mm pieces and digested with 1 mg/mL of collagenase type 1A in DMEM/F12 for 1.5 h at 37°C. It was then filtered through 250 µm nylon sieve to remove mucus and undigested tissue. The filter was passed through a 40 µm nylon sieve, which allowed the stromal cells to pass through, while intact glands were retained. Glands were recovered from the filter by backwashing with DMEM/F12 containing 10% bovine serum albumin and seeded on to the six-well plates for future study for certain durations.

The Ishikawa cell line (American Type Culture Collection, Manassas, VA, USA) was obtained from Shanghai Institutes for Biological Science and maintained in RPMI-1640 medium (Gibco; Thermo Fisher Scientific) containing 10% fetal bovine serum (FBS) and 100 U/mL penicillin and streptomycin antibiotics. When the cells reached confluence, the medium was replaced with phenol red free RPMI-1640 supplemented with 10% charcoal/dextran-treated FBS (all purchased from Sigma-Aldrich). For hormonal treatments, E_2_ (Sigma-Aldrich) was added to the culture media to a final concentration of 10^−9^ M (close to the physiological concentration in women at the mid-secretory phase) and 10^−7^ M (close to the supraphysiological concentration in women with controlled ovarian hyperstimulation), for certain durations according to the experimental purposes (Valbuena *et al*. 2001).

### JAr spheroid attachment to endometrial epithelial cell monolayers

In *in vitro* attachment model, multicellular spheroids of human choriocarcinoma (JAr) cells (American Type Culture Collection, Manassas, VA, USA; HTB 144) were applied to receptive-phase human endometrial epithelial cell monolayers and Ishikawa cell layers, respectively. The endometrial epithelial cells were pretreated with 10^−9^ M or 10^−7^ M E_2_ and cultured for 3 days. JAr spheroids were prepared according to a standard procedure (Hohn *et al*. 2000) and transferred onto the surface of confluent cell monolayers for 1 h (50 spheroids/dish for primary cultured endometrial cells and 45 spheroids/dish for Ishikawa cells). Non-adherent spheroids were detached by centrifugation (10 ***g***; 10 min) of the six-well plates with the cell surface facing down. We counted the attached spheroids under a light microscope and the attachment rate was calculated for each well as follows: attachment rate equals the ratio of the number of spheroids attached to the number of spheroids seeded. This experiment was repeated at least three times.

### Protein extraction

Protein extractions from Ishikawa cells treated with 10^−9^ M and 10^−7^ M E_2_, respectively, were performed with cell lysis buffer (4% SDS, 1 mM DTT, 150 mM Tris–HCl, pH 8.0) at 95°C for 20 min, followed by sonication on ice. The crude extracts were incubated at 95°C for 5 min and cleared by centrifugation (SCILOGEX D3024R, Inc., Rocky Hill, CT, USA) at 14,000 ***g*** for 30 min at 15°C. Thereafter, the supernatant was collected and protein concentration was measured by the BCA protein assay reagent (Pierce).

### Protein digestion and iTRAQ labeling

Protein digestion was performed according to the FASP procedure (Wisniewski *et al*. 2009). Four biological replicates were included in the analysis. Briefly, 200 μg of total protein samples were diluted in 30 μL 4% SDS, 100 mM Tris–HCl pH 8.0 and 100 mM dithiothreitol solution and heated at 95°C for 5 min. After each sample was cooled to room temperature, it was loaded onto an ultrafiltration filter (cutoff 10 kDa, Sartorius, Germany). We added 200 μL UT buffer (8 M Urea and 150 mM Tris–HCl, pH 8.0) to the filter and centrifuged it at 14,000 ***g*** at 20°C for 30 min. Subsequently, 100 μL of iodoacetamide solution (50 mM iodoacetamide in UT buffer) was added for blocking reduced cysteines, and the samples were further incubated for 20 min in darkness. The filters were centrifuged at 14,000 ***g*** at 20°C for 20 min and washed (twice) with 100 μL UT buffer at 14,000 ***g*** for further 20 min. The dissolution buffer (100 μL, AB Sciex, Framingham, MA, USA) was added to the filter followed by centrifugation at 14,000 ***g*** at 20°C for 30 min. This step was repeated twice. Finally, 40 μL of trypsin (Promega) buffer (2 μg trypsin in 40 μL dissolution buffer) was added, and the samples were digested overnight at 37°C. Each filter unit was transferred to a new tube and centrifuged at 14,000 ***g*** at 20°C for 30 min. The resulting peptide concentrations were estimated by UV light spectral density at OD280 (Sheng *et al*. 2012). Then, the peptide mixtures were labeled using the 8-plex iTRAQ reagent according to the manufacturer’s instructions (AB Sciex, Framingham). Four samples from control group, treated with 10^−9^ M E_2_, were labeled with mass 114, 115, 116 and 117 isobaric iTRAQ tags, while the other four samples from high E_2_ group (10^−7^ M E_2_) were labeled with mass 118, 119, 120 and 121 isobaric iTRAQ tags. The labeling solution was incubated at room temperature for 2 h before further analysis.

### Strong cationic exchange chromatography separation

The combined sample was acidified (pH = 8.0) with 1% trifluoroacetic acid before being subjected to strong cationic-exchange chromatography (SCX) fractionation using a PolySULFOETHYL column (4.6 × 100 mm, 5 μm, 200 Å, Poly LC Inc., Columbia, MD, USA). Solvent A consisted of 10 mM KH_2_PO_4_ in 25% (v/v) ACN and solvent B was solvent A with 500 mM KCl added. The solvents were applied using a gradient of 0–10% solvent B for 2 min, 10–20% solvent B for 25 min, 20–45% solvent B for 5 min and 50–100% solvent B for 5 min. The elution was monitored by absorbance at 214 nm and fractions were collected every 1 min. Finally, these samples were combined into 10 fractions based on the quantity of peptide and then desalted on C18 cartridges (Sigma). Each SCX salt step fraction was dried in a vacuum centrifuge and reconstituted with 40 μL 0.1% (v/v) trifluoroacetic acid.

### LC–ESI-MS/MS analysis

Peptide mixture (5 μg) from each fraction was subjected to nano LC–MS/MS analysis. The mixtures were loaded onto the Thermo EASY-nLC column (Thermo Finnigan, San Jose, CA, USA) (100 mm × 75 μm, 3 μm) in solvent C (0.1% formic acid) and separated with a linear gradient of solvent D (80% acetonitrile with 0.1% (v/v) formic acid) at a flow rate of 300 nL/min over 120 min: 0–100 min with 0–45% solvent D; 100–108 min with 45–100% solvent D and 108–120 min with 100% solvent D. The Q-Exactive (Thermo Finnigan) mass spectrometer acquired data in the positive ion mode (2.2 kV) with a selected mass range of 300–800 mass/charge (m/z). Dynamic exclusion was used with 40.0 s duration. Q-Exactive survey scans were set as 70,000 at m/z 200 and 17,500 at m/z 200 of resolution for HCD spectra. MS/MS data were acquired using a data-dependent acquisition method with the top 10 most abundant precursor ions. The normalized collision energy was 30 eV and the under fill ratio was defined as 0.1% on the Q-Exactive.

### Protein identification and quantification

Protein identification and quantification were performed with high accuracy using MaxQuant, version 1.2.2.5 software in combination with Andromeda search engine (Cox *et al*. 2011). The acquired data from triplicate MS runs for each sample were combined and searched against an International Protein Index (IPI 3.83) human protein sequence database using the MaxQuant computational proteomics platform, version 1.2.0.18 (Cox & Mann 2008). A decoy version of the IPI human database was used to estimate peptide and protein false discovery rate. A FDR of 0.01 was applied for both protein and peptide identification, ensuring that at most only 1% of proteins would be falsely identified. Ratios were obtained for both the groups 10^−9^ M to 10^−7^ M E_2_ and then inversed. Significant protein ratio, cutoff, was set at a significance *B* value ≤0.05 as calculated by MaxQuant (Cox & Mann 2008, Cox *et al*. 2009). Carbamidomethylation of cysteine was set as a fixed modification, with protein N-terminal acetylation and oxidation of methionine as variable modifications, enzyme: trypsin/P, maximum number of missed cleavages. The processed MS data generated by MaxQuant are presented in the supporting information data Table 1.

**Table 1:** Estrogen-induced differentially expressed proteins in human endometrial epithelial cells identified from iTRAQ analysis.

P value	**Fold change**	**Accession code**	Protein description	Score	**Cover-age** (%)	**Mol. Wt** (kDa)	**Unique peptides**	Gene symbol
1.69E-02	−1.47	E5RK39	Ribonucleases P/MRP protein subunit POP1	2.8711	10.4	20.7	2	POP1
3.27E-02	−1.34	Q9Y6K0	Choline/ethanolaminephosphotransferase 1	3.1822	4.8	46.553	2	CEPT1
3.59E-02	1.3	Q9NZ08	Endoplasmic reticulum aminopeptidase 1	12.72	5.2	107.23	3	ERAP1
2.83E-02	1.3	P10412	Histone H1.4	17.536	48.4	21.865	4	HIST1H1E
4.18E-02	1.31	P01044	Kininogen-1	26.763	9.7	68.964	5	KNG1
2.37E-03	1.31	Q9C0J8	Pre-mRNA 3ʹ end processing protein WDR33	5.6023	2.7	145.89	2	WDR33
4.03E-02	1.31	P06868	Plasminogen	17.058	6.4	91.215	5	PLG
1.66E-02	1.31	I3L0N3	Vesicle-fusing ATPase	70.446	24.1	82.091	16	NSF
4.74E-03	1.31	Q9UH99	SUN domain-containing protein 2	85.535	16.1	79.085	7	SUN2
1.31E-02	1.32	Q28085	Complement factor H	4.3809	1.2	140.37	2	CFH
4.35E-04	1.32	Q3MHN2	Complement component C9	139.78	16.2	61.998	6	C9
4.80E-02	1.33	Q71DI3	Histone H3.2	14.879	28.7	15.388	1	HIST2H3A
2.63E-03	1.34	Q0VCM5	Inter-alpha-trypsin inhibitor heavy chain H1	52.463	5.4	101.24	3	ITIH1
1.13E-02	1.35	Q3KUS7	Complement factor B	24.379	5.9	85.411	4	BF
1.45E-02	1.35	P02070	Hemoglobin subunit beta	137.67	68.3	15.954	9	HBB
4.00E-02	1.35	P01966	Hemoglobin subunit alpha	87.333	66.9	15.184	8	HBA
3.11E-02	1.37	J3KMX2	SWI/SNF-related matrix-associated actin-dependent regulator of chromatin subfamily D member 2	4.5084	8.3	52.238	2	SMARCD2
1.71E-02	1.37	E9PH82	Protein FAM98A	50.655	20.8	34.431	4	FAM98A
3.34E-02	1.39	Q9UDR5	Alpha-aminoadipic semialdehyde synthase, mitochondrial	5.4278	3.9	102.13	3	AASS
8.02E-03	1.4	E9PJ95	COMM domain-containing protein 9	12.306	11.4	20.687	1	COMMD9
3.77E-03	1.51	Q3SZV7	Hemopexin	19.7	11.1	52.295	5	HPX
1.03E-02	1.52	P50448	Factor XIIa inhibitor	37.23	8.8	51.723	3	N/A
6.31E-05	1.52	P21752	Thymosin beta-10	38.232	33.3	4.8054	2	TMSB10
3.91E-04	1.53	Q3MHN5	Vitamin D-binding protein	16.019	9.1	53.341	4	GC
2.78E-03	1.54	Q05B55	IGK protein	5.2815	7.9	26.59	2	IGK
2.63E-04	1.55	A2I7N3	Serpin A3-7	4.3669	10.1	46.941	2	SERPINA3-7
8.23E-04	1.59	Q2UVX4	Complement C3	323.31	33.9	187.37	48	C3
3.06E-03	1.6	Q9UBI6	Guanine nucleotide-binding protein G(I)/G(S)/G(O) subunit gamma-12	12.514	47.2	8.0061	3	GNG12
1.52E-03	1.62	B0YIW2	Apolipoprotein C-III	94.585	13.7	12.815	1	APOC3
2.07E-03	1.63	B9A064	Immunoglobulin lambda-like polypeptide 5	2.1299	3.7	23.063	1	IGLL5
3.80E-04	1.65	Q3bib52	Inter-alpha-trypsin inhibitor heavy chain H4	29.331	9.6	101.51	9	ITIH4
5.03E-04	1.68	P12763	Alpha-2-Heremans Schmidt (HS) glycoprotein	21.332	14.2	38.418	5	AHSG
1.96E-03	1.71	P17697	Clusterin	2.0487	2.3	51.113	1	CLU
1.29E-03	1.78	Q9TRI1	Inter-alpha-trypsin inhibitor HC2 component homolog	35.657	4.7	106.19	3	N/A
1.09E-04	1.92	Q9TTE1	Serpin A3-1	132.16	21.7	46.203	3	SERPINA3-1
1.34E-04	1.97	P15497	Apolipoprotein A-I	185.73	61.1	30.276	16	APOA1
3.22E-04	2.02	Q2KIT0	Protein HP-20 homolog	9.9797	13.1	20.646	2	N/A
2.50E-04	2.06	A2I7N0	Serpin A3-5	39.18	17.8	46.397	2	SERPINA3-5
1.05E-04	2.09	Q29443	Serotransferrin	138.25	28.7	77.738	16	TF
1.54E-04	2.09	P81644	Apolipoprotein A-II	14.097	35	11.202	3	APOA2
1.65E-04	2.1	Q3SZR3	Alpha-1-acid glycoprotein	60.603	30.7	23.182	6	ORM1
1.83E-04	2.19	Q1RMN8	Immunoglobulin light chain, lambda gene cluster	27.788	17.9	24.536	3	IGL@
6.33E-05	2.3	P02769	Serum albumin	323.31	30.6	69.293	21	ALB
1.26E-03	2.47	P43366	Melanoma-associated antigen B1	1.785	2.6	39.037	1	MAGEB1
2.79E-04	2.84	Q2KJF1	Alpha-1B-glycoprotein	43.931	11.7	53.553	4	A1BG

### Western blotting analysis

The cell extracts were prepared by lysing unsorted epithelial cells with RIPA buffer containing 150 mM NaCl, 50 mM Tris–HCl (pH = 8), 1% NP-40, 0.5% sodium deoxycholate, 0.1% SDS, protease inhibitors and phosphatase inhibitors (Sigma). The cell extract (20 µg) was run on an 8–10% SDS-PAGE gel and transferred to a nitrocellulose transfer membrane (Bio-Rad). After incubating for 1 h with blocking buffer, the membrane was incubated overnight at 4°C with mouse monoclonal anti-complement C_3_ (1:500 Santa Cruz Biotechnology sc28294), mouse monoclonal anti-plasminogen (1:500 Santa Cruz Biotechnology sc376324), rabbit polyclonal anti-kininogen 1 (1:1000 Abcam ab97761) and mouse polyclonal anti-GAPDH antibody (1:5000 Novus Biologicals, Littleton, CO, USA). After three washes with 1× TBST, pH 7.4, the samples were then incubated with fluorescence-labeled anti-mouse IgG or anti-rabbit IgG antibody (Daylight 680 or 800, KPL; 1:5000) for 1 h at room temperature and analyzed with an Odyssey Imager (Li-Cor; Lincoln, NE, USA).

### Immunofluorescence analysis

Human endometrial samples at the receptive phase (LH + 7) were used for immunofluorescence validation. Samples were fixed in 10% formalin and processed for paraffin embedding. Cross-sections (5 µm thickness) were mounted onto microscope slides (Thermo Fisher Scientific). After deparaffinization and rehydration, sections were rinsed three times with phosphate buffered saline (PBS) for 5 min. Immunofluorescence analysis was performed on endometrial sections using the LSAB Peroxidase Kit (DAKO). Non-specific binding was blocked with 5% bovine serum albumin (BSA). Sections were incubated with the following primary antibodies diluted (1:100) in blocking solution (0.25% BSA, 0.3% Triton X-100, sterile PBS) overnight at 4°C. Plasminogen (Abcam 154560), Kininogen 1 (Abcam ab97761), ERα (Abcam ab108398) and C_3_ (Proteintech, 21337-1-AP, Chicago, IL, USA). Tissue sections were then washed with PBS for 5 min. For the fluorescent detection (anti-plasminogen, anti-kininogen 1, anti-C_3_ and anti-ERα), Alexa Fluor 488 goat anti-rabbit (dilution 1:100, Thermo Fisher Scientific) secondary antibody was used and nuclear counterstaining was performed with 4,6-diamidino-2-phenylindole (DAPI, Molecular Probes/Life Technologies). Evaluation of the sections was performed using confocal laser scanning microscopy (Zeiss 800, LSM 510 Meta).

### Bioinformatics analysis

Differentially expressed protein profiles (*P* <0.05) were selected and the ones with differential expression ratio of over ±1.2 were retained. The capability of the resulting differentially expressed proteins in differentiating two groups of samples was then evaluated by hierarchical cluster analysis. For this purpose, the Cluster 3.0 (http://bonsai.hgc.jp/~mdehoon/software/cluster/software.htm) and the Java Tree view software (http://jtreeview.sourceforge.net) were used. Disease analysis, pathway and network generation were performed using IPA software package (QIAGEN). IPA is a knowledge database relying on published literature related to protein function, localization, relevant interactions and biological mechanisms. Calculated the *z*-score can infer the activation states (‘activated’ or ‘inhibited’) of implicated biological processes.

### Statistical analysis

GraphPad Prism 6 (GraphPad Software) was used for statistical analysis. Fisher’s exact test was used to calculate a *P* value to determine the probability that the association between proteins in the dataset, and, the biological process could be explained by chance alone. Using Student (unpaired) *t*-test, statistical significance for comparison between two groups was determined. Four biological replicates were tested for all samples and the data are expressed as means ± s.d. *P* < 0.05 was considered significant.

### Ethics approval and consent to participate

The study was approved by the ethical committee of Zhejiang University, Hangzhou 310058, China. Written informed consent for study participation was obtained from the participants.

## Results

### Effects of E_2_ at different concentrations on JAr spheroid attachment to human endometrial epithelial cell monolayers

**Figure 1:**
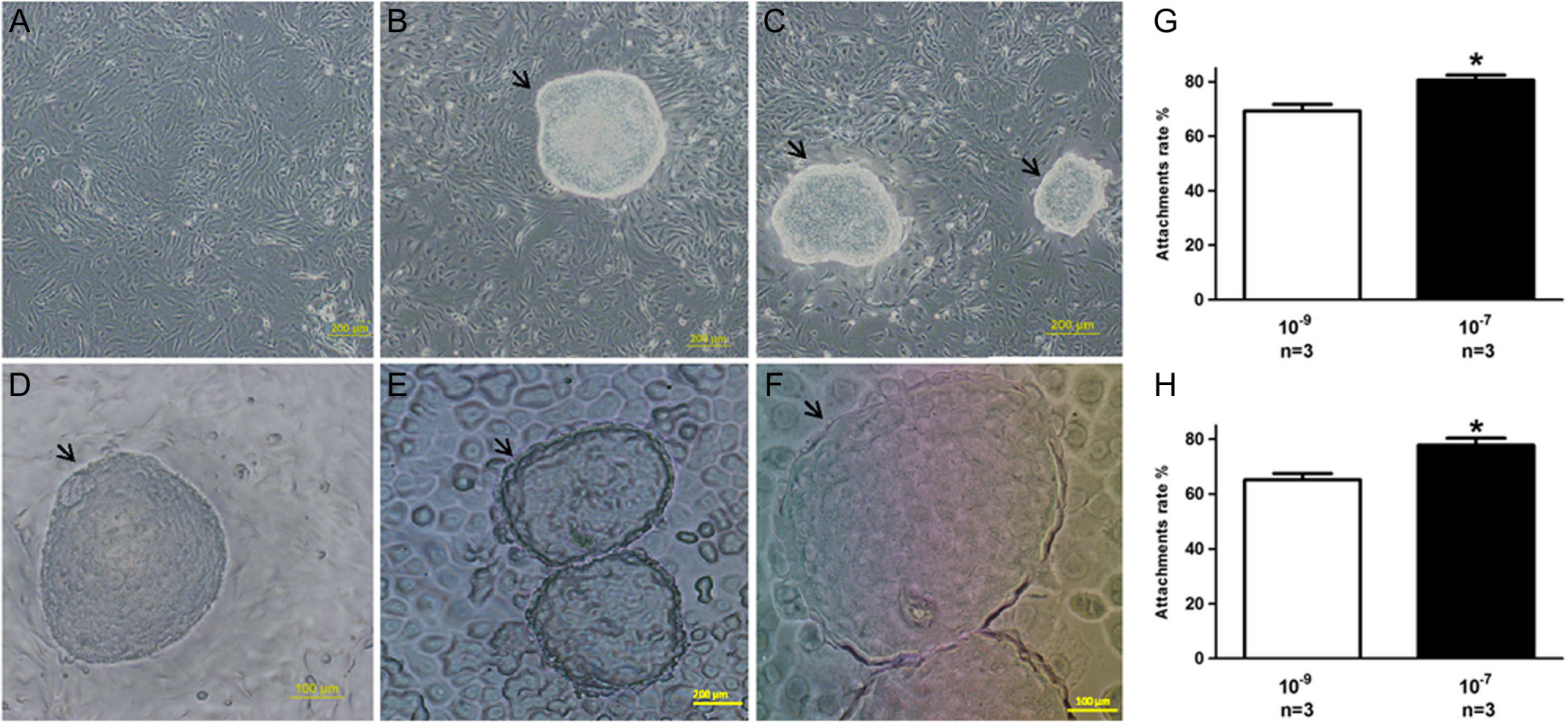
Effect of E_2_ on JAr adhesion in treated human endometrial epithelial cells and Ishikawa cells. (A) Receptive-phase human endometrial epithelial cells treated with different E_2_ concentrations. (B) and (C) Attachment of JAr spheroids to 10^−9^ M E_2_- and 10^−7^ M E_2_-treated cells, respectively (magnification 10×). (D) JAr spheroids attachment (magnification 20×). (E) and (F) JAr spheroids attachment to the Ishikawa cell monolayers (magnification 10×; 20×). Arrow indicates JAr spheroids. (G) and (H) Attachment rate of Jar spheroids to human endometrial epithelial cells and Ishikawa cells, respectively. Data are present as mean ± s.d. *n* = the times of experiment repeated. *P < 0.05 compared with control. A full colour version of this figure is available at http://dx.doi.org/10.1530/JME-17-0036

To clarify whether treatments of human endometrium with different estrogen concentrations affected embryo implantation, we used *in vitro* attachment model of human choriocarcinoma JAr cell spheroids to receptive-phase endometrial epithelial cell monolayers and Ishikawa cell monolayers (Fig. 1A, B, C, D, E and F). The attachment rate of JAr spheroids to the endometrial cells pretreated with 10^−9^ M E_2_ for 3 days was 69.33 ± 2.40%. However, the attachment rate was enhanced to 80.67 ± 1.76% after treatment of the epithelial cells with 10^−7^ M E_2_ (Fig. 1G). On the other hand, the attachment rate of JAr spheroids to the Ishikawa cells pretreated with 10^−9^ M E_2_ for 3 days was 65.1 ± 2.08% and the attachment rate was also enhanced to 77.5 ± 2.56% after treatment of the Ishikawa cells with 10^−7^ M E_2_ (Fig. 1H). These results suggest that, compared to 10^−9^ M E_2_, 10^−7^ M E_2_ may improve the endometrial receptivity.

### Protein expression profiles in cells treated with different concentrations of E_2_

**Figure 2:**
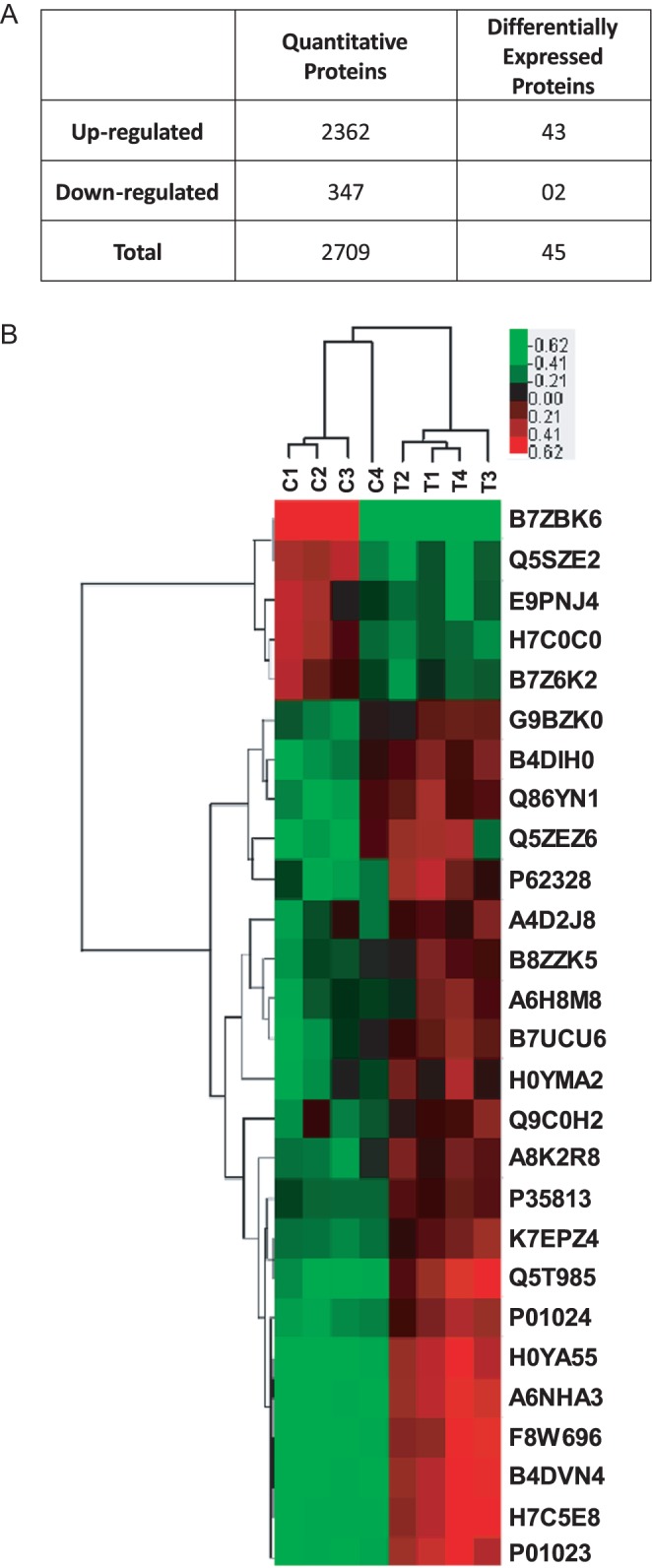
Altered protein profiles identified in Ishikawa cells. (A) The number of identified proteins including both upregulated and downregulated proteins. (B) Hierarchical clustering of differentially expressed protein profiles (red: upregulated proteins; green: downregulated proteins; black portrays: no change). Bar color represents a logarithmic scale from −0.62 to +0.62.

The iTRAQ analysis was performed to identify proteome changes in samples of Ishikawa cells treated with different E_2_ concentrations (10^−9^ M or 10^−7^ M). Protein identification and quantification from the four biological replicates were subjected to LC–MS/MS and MaxQuant (MQ) (1.2.2.5) analysis. According to the UniProtKB *Homo sapiens* reference proteome database containing 70,136 canonical and isoform sequences through MaxQuant’s built-in Andromeda search engine, a total of 2709 cellular proteins were considered to be statistically significant following exclusion, including 2362 upregulated proteins and 347 downregulated proteins (Fig. 2A). A FDR was applied combined with filtering out those ratios with a significance of *B* value ≤0.05. Compared with the control group (10^−9^ M E_2_), 45 differentially expressed proteins in Ishikawa cells treated with 10^−7^ M E_2_ were identified, containing 43 upregulated and 2 downregulated proteins (Table 1). The hierarchical clustering of these differentially expressed protein profiles are visualized in a heat map (Fig. 2B). Results showed a striking separation of the two groups into two major opposing branches, indicating that the proteins expressed in 10^−7^ M E_2_-treated group were distinct from those treated with 10^−9^ M E_2_.

### Validation of differentially expressed proteins by Western blot analysis

**Figure 3:**
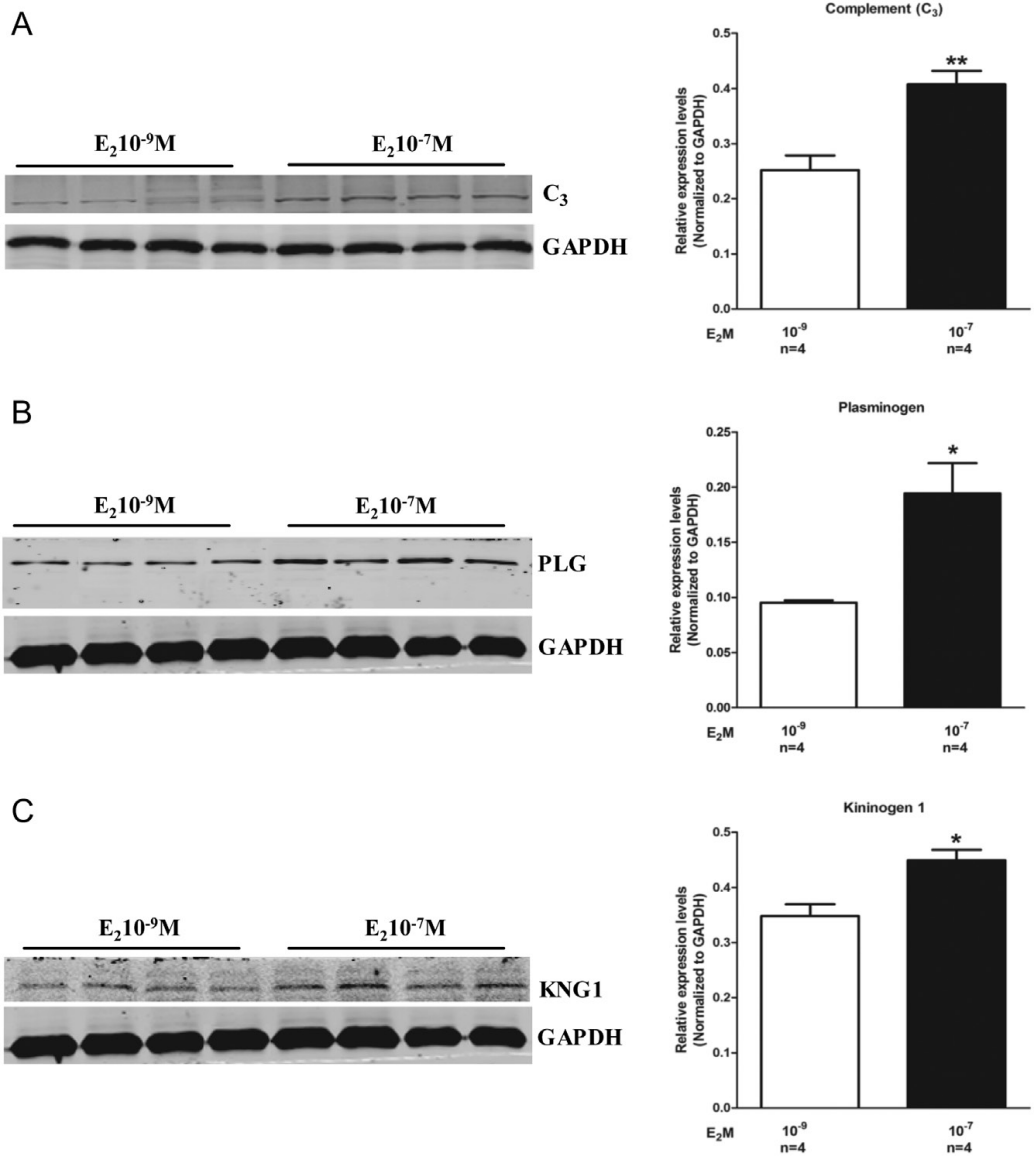
Differential expression of (A) C_3_, (B) plasminogen and (C) kininogen-1 by Western blot. Data are present as mean ± s.d. (*n* = 4). * and **, *P* < 0.05 and *P* < 0.01 compared with the corresponding control, respectively.

To confirm the proteomic results, three upregulated proteins with known roles in embryo adhesion including complement (C_3_), plasminogen and kininogen-1 were validated by Western blot analysis in both the groups. The results revealed that C_3_, plasminogen and kininogen-1 were indeed highly expressed in Ishikawa cells treated with 10^−7^ M E_2_ (Fig. 3A, B and C). These results were essentially in agreement with those of proteomic analysis, suggesting that 10^−7^ M E_2_ might increase C_3_, plasminogen and kininogen-1 expression levels in endometrial cells, which in turn might promote the adhesion of JAr spheroids to the human endometrial cell monolayers.

### Localization of C_3_, KNG1, PLG and ERα in the receptive endometrium

**Figure 4:**
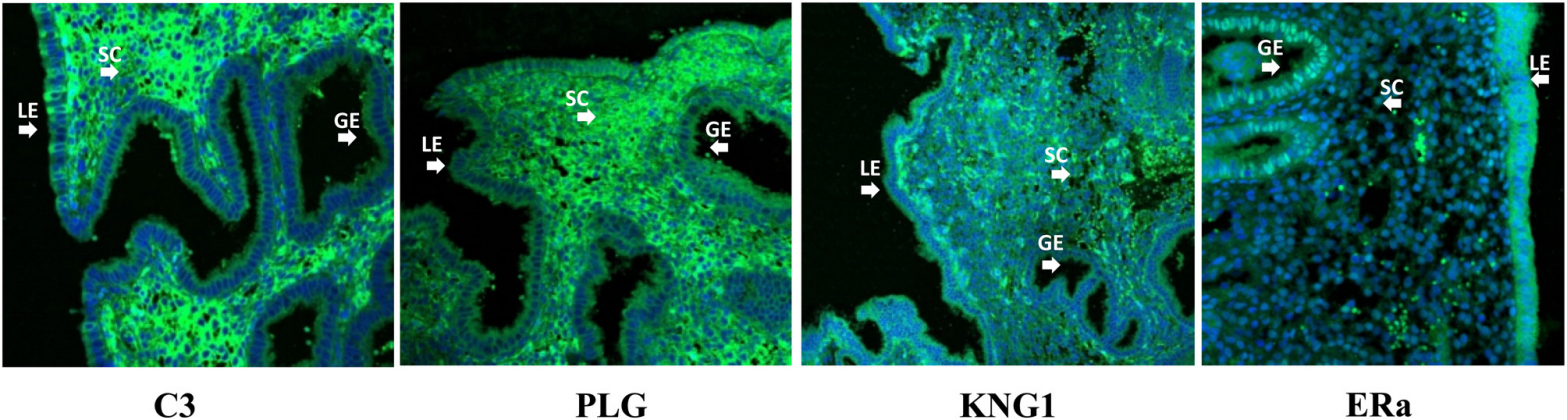
Localization of protein expression (C_3_, KNG1, PLG and ERα) in luminal epithelium (LE), glandular epithelium (GE) and stromal cells (SC) was examined in the human endometrium at the receptive phase by immunofluorescence (×200). Green: C_3_, KNG1, PLG and ERα staining. Blue: nuclei counterstained with DAPI.

As C3, KNG1, PLG and ERα (alpha) have been shown to have major roles in endometrial receptivity, we analyzed the location of these proteins in the human endometrium at the receptive phase by immunofluorescence. The staining (green) of C3 and PLG was shown in luminal epithelium (LE) and glandular epithelium (GE), largely restricted to apical surface of luminal and glandular epithelial cells. Furthermore, stromal cells (SC) also expressed higher density of C3 and PLG at the receptive phase. KNG1 and ERα followed the opposite trend, with a stronger intensity (green) in the LE and GE apical surfaces in the receptive endometrium (Fig. 4).

### Ingenuity pathway analysis (IPA) yielded distinct functional groupings

**Figure 5:**
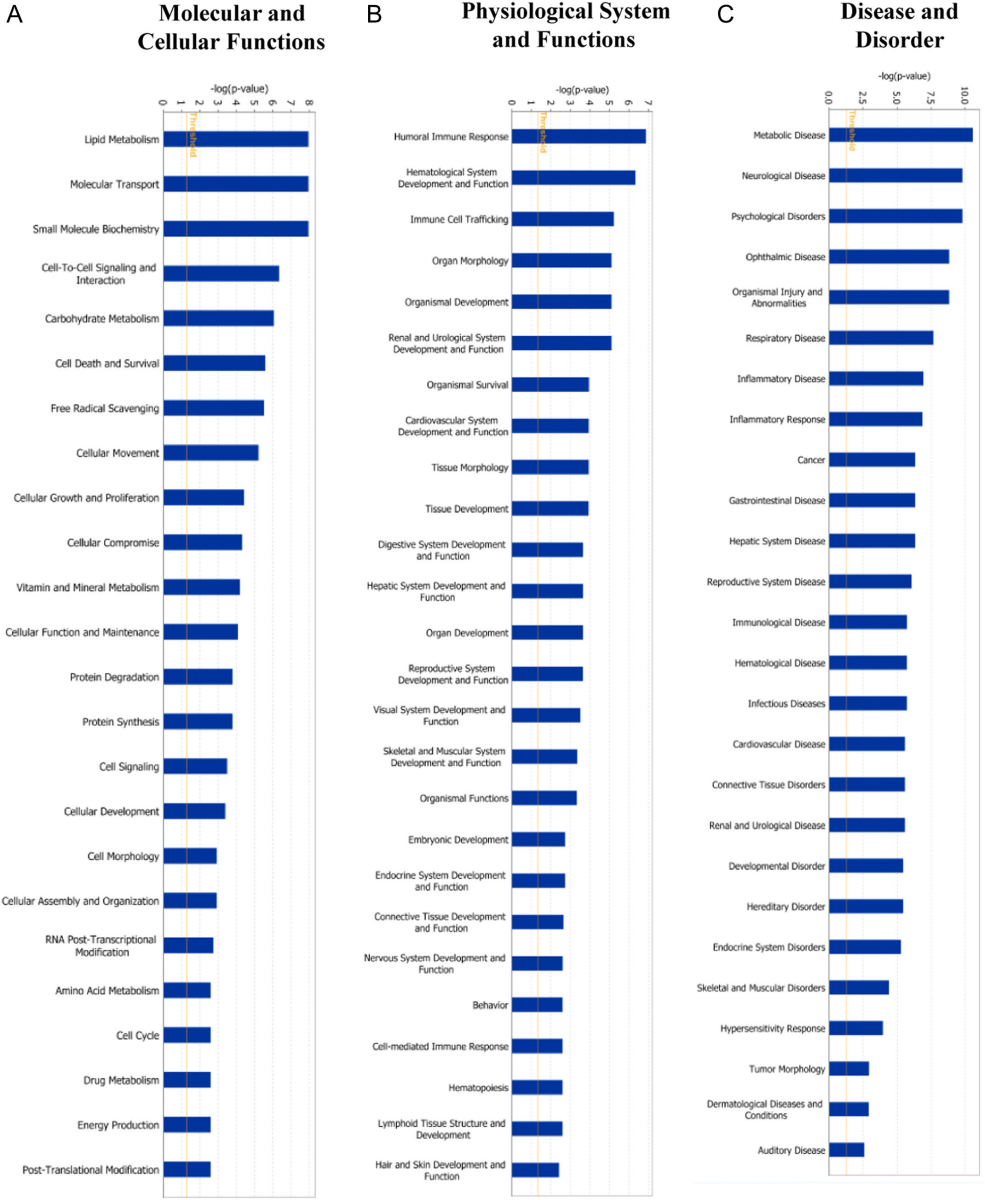
IPA-based functional analysis of 45 differentially expressed protein profiles. (A) Molecular and cellular functions; (B) physiological system and functions; (C) disease and disorder. A full colour version of this figure is available at http://dx.doi.org/10.1530/JME-17-0036

**Figure 6:**
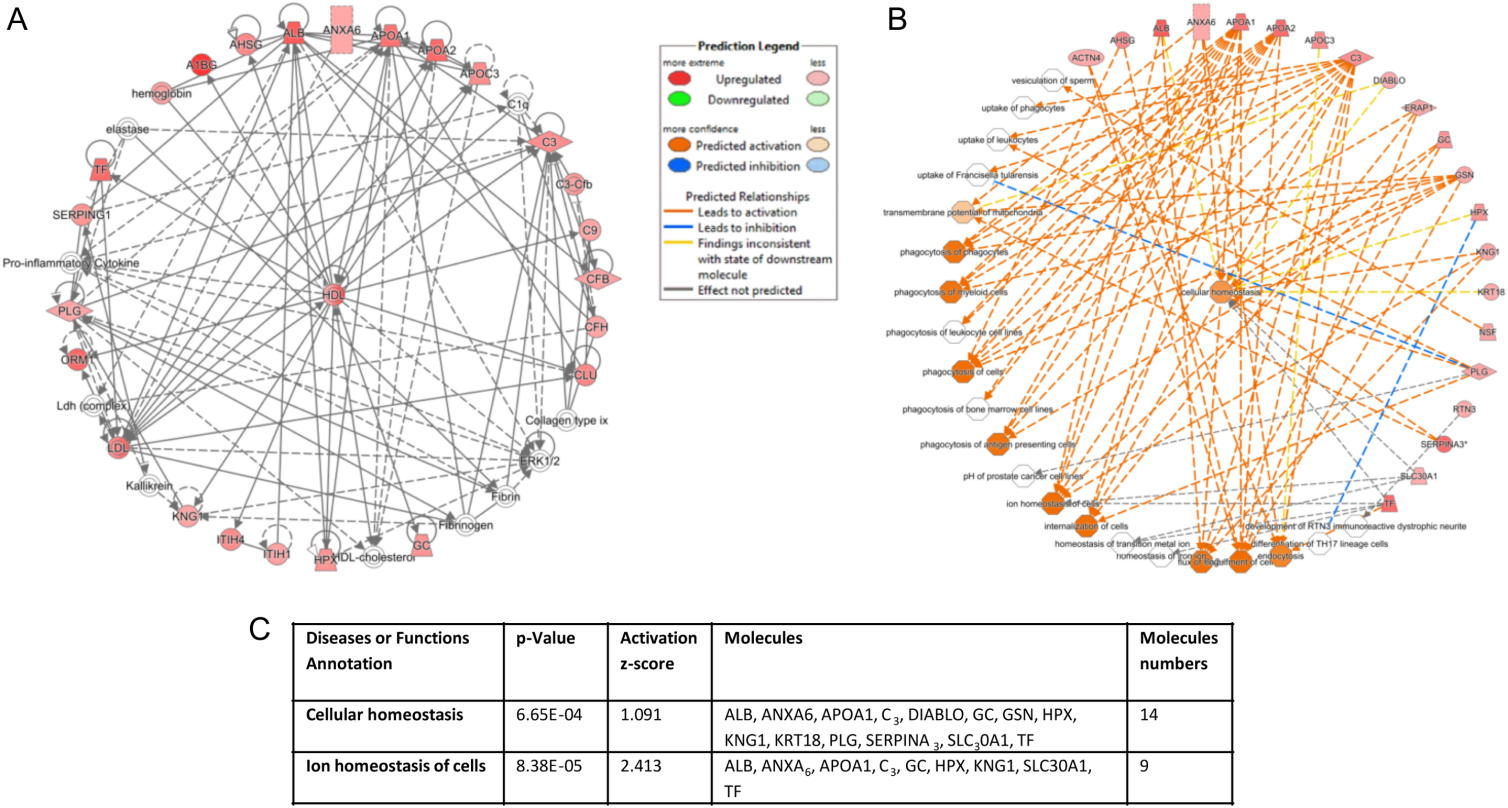
(A) Top connectivity network among differentially expressed protein profiles. (B) Downstream effect analysis within functional group ‘cellular function and maintenance’. The protein names and their temporal expression are shown in Table 1. Different color lines connecting proteins represent the relationships between them in Ishikawa cells (red coloration indicates an increase in expression, green indicates a decrease in expression and lack of color indicates a protein involved in bridging interactions, but was not identified as differentially regulated). The networks were generated through the use of IPA, genes or gene products are represented as nodes and the biological relationship between two nodes is represented as an edge. (C) Protein names and their temporal expression are listed in the table.

IPA was applied to analyze the relationship among 45 differentially expressed protein profiles based on their interaction and function. The results showed that most of the differentially expressed proteins in human endometrial epithelial cells treated with E_2_ (10^−7^ M) were associated with organization of ‘molecular and cellular functions’ (Fig. 5A), ‘physiological system and functions’ (Fig. 5B) and ‘disease and disorder’ (Fig. 5C). Based on overlying *P* values, differentially expressed proteins in both organization of ‘physiological system and functions’ and ‘disease and disorder’ were related significantly with 26 subcategories separately, while 24 subcategories were linked to ‘molecular and cellular functions’. To understand the specific interaction of proteins that showed significant changes within functional groupings, we examined interaction networks generated by IPA (Fig. 6A). In ‘molecular and cellular functions’ classification, further downstream effect analysis within functional groupings of ‘cellular growth and maintenance’ revealed that the differentially expressed proteins were mainly associated with cellular homeostasis (Fig. 6B and C).

### Canonical signaling pathways of differentially expressed proteins

IPA, a web-based tool, also highlighted two canonical signaling pathways, including the LXR/RXR activation pathway and the acute-phase response signaling pathway mediated by the greatest number of identified differentially expressed proteins along with a *z*-score representing the log probability being found by random chance. The differentially expressed proteins in endometrial cell lines were predominantly associated with the regulating pathways suggesting that they might have a critical role in embryo adhesion (Table 2).

**Table 2:** IPA-defined canonical pathways for differentially expressed proteins identified by iTRAQ.

**Ingenuity canonical pathways**	Log (P-value)	z-score	Molecules	Top functions and diseases
LXR/RXR Activation	2.11E + 01	3.873	KNG1, HPX, C3, APOA2, AHSG, C9, A1BG, ALB, APOA1, TF, ORM1, ITIH4, GC, CLU, APOC3	Lipid absorption, storage, and utilization
Acute-phase response signaling	1.72E + 01	1.633	PLG, ALB, HPX, SERPING1, APOA1, C3, ORM1, TF, ITIH4, APOA2, C9, AHSG, CFB, SERPINA3	Defense mechanism against inflammation

### Upstream analysis

Upstream regulator analysis may predict upstream molecules, including transcription factor, microRNA, kinase, compound or drug, which may cause the altered protein expression patterns. The results in Table 3 provided a list of upstream regulators related to embryo implantation that were predicted to be activated or inhibited based upon the activation *z*-score for uploaded differentially expressed protein profiles.

**Table 3:** IPA-based upstream analysis for 10^−7^ M E_2_ induced differentially expressed proteins associated with implantation.

**Upstream regulator**	Molecule type	Predicted state	Activation ***z*-score**	***P* value of overlap**	**Target molecules in dataset**	Finding(s)
TNF	Cytokine	Activated	2.001	2.79E-02	ALB, APOA1, C3, CFB, CLU, ORM1, SERPINA3, TF	In uterus of female TNFα cyclic expression causes proliferation of numerous types of cells (Kopf *et al*. 1994, Tabibzadeh *et al*. 1995)
IL6	Cytokine	Activated	2.36	4.06E-06	ALB, APOA1, C3, CLU, HPX, KRT-18, ORM1, PLG, SERPINA3, TF	Cyclic expression of IL-1, IL-6 mediate cells proliferation in female uterus. IL-6 deficient (−/−) mice showed reduced implantation sites, impaired immune and acute-phase responses (Kopf *et al*. 1994, Tabibzadeh *et al*. 1995)
Hmgn3	Other	Activated	2	8.04E-07	AHSG, APOA1, APOA2, KNG1	Hmgn3 modulate Hand2 gene expression in uterine cells (Okada *et al*. 2014). Hand-2 deficient (−/−) gene led to implantation failure in mice (Okada *et al*. 2014)
miR-140-3p	Mature-microRNA	Inhibited	−2.236	3.73E-02	AASS, BCAS2, ERAP, NSF, WDR33	miR-140-3p affect expression of putative targets in endometrial stromal cells *in vitro* (Okada *et al*. 2014)

## Discussion

Embryonic implantation in humans depends on the interaction of the embryo with the receptive endometrium. Exposure to high levels of E_2_ during COH in the early follicular phase is related to a lower chance of pregnancy and IVF outcome (Kolibianakis *et al*. 2003). Other investigators directed that supraphysiological E_2_ levels were not detrimental to IVF outcome (Levi *et al*. 2001, Peña *et al*. 2002). Kolb and Paulson (1997) reported that, in COH, exposure to peak levels of E_2_ in the early luteal phase could lead to a time shift of the implantation window. Joo and coworkers (2010) demonstrated that optimizing levels of serum E_2_ improved pregnancy outcome of *in vitro* fertilization in a concentration-dependent fashion during COH cycles. However, the enigma is still unclear. To clarify this issue and to examine the endometrial response to serum E_2_ levels, we designed the present study for the first time to compare and quantify the differentially expressed protein profiles in Ishikawa cells treated with different regimen of estrogen (10^−9^ M and 10^−7^ M). We found that, compared to physiological concentration of E_2_ (10^−9^ M), supraphysiological E_2_ concentration (10^−7^ M) significantly increased the attachment rates of JAr spheroids to both human endometrial epithelial cell monolayers and Ishikawa cell monolayers. We evaluated that majority of proteins showed a significantly higher response to 10^−7^ M E_2_ compared to 10^−9^ M E_2_. Several proteins such as C_3_, SERPINs, plasminogen, kininogen-1, endoplasmic reticulum aminopeptidase-1 and alpha-1-acid glycoprotein have been detected to be involved in endometrial receptivity. Previous efforts identified that E_2_ administration stimulated C_3_ synthesis in uterine luminal epithelial cells of rats (Sundstrom *et al*. 1989), whereas progesterone blocked induction of cell proliferation and C_3_ synthesis in the epithelium of rat uterus (Bigsby 1993). In glandular epithelial cells of rat luteal endometrium C_3_ synthesis was upregulated by estrogen (Hasty *et al*. 1994). C_3_ expression is also modulated by hCG in human endometrial compartments during the implantation window (Palomino *et al*. 2013). We detected high levels of C_3_ in Ishikawa cells after 10^−7^ M E_2_ treatment, suggesting a significantly endometrial response to a reasonable serum E_2_ level in controlled ovarian stimulation. The data from the present study also demonstrated significant expression of C_3_ in the receptive-phase endometrium. Plasminogen activators (PA) in human endometrium play an important role in pathophysiological aspects of tissue expansion and remodeling. Ovarian hormonal patterns affect cyclic expression of the activity of plasminogen activators, predominantly t-PA. Estrogen stimulates PA synthesis while progesterone declines their synthesis (Koh *et al*. 1992). In normal endometrium, cyclic variation and distribution of uPA, uPAR and plasminogen activator inhibitor 1 (PAI-1) have been reported with discordant levels of uPA in both proliferative and secretory phases, while uPAR only in the secretory phase (Nordengren *et al*. 2004). In our present findings, plasminogen and a number of potent SERPINs that modulate the proteolytic activities of PA (tPA/uPA) (Lee *et al*. 2011) have been determined to be significantly upregulated in the endometrial cells during 10^−7^ M E_2_ treatment. Kallikrein–kinin system has a key role in many inflammatory processes during proliferation of the endometrial lining of uterus (Clements *et al*. 1997). The present findings revealed high expression levels of kininogen-1 in human endometrial epithelial cells after 10^−7^ M E_2_ treatment, indicating that kallikrein–kinin system may be activated. Interestingly, the distribution and localization of PLG and KNG1 in the human endometrial compartments at the receptive phase of the cycle reflects their involvement in endometrial preparations for implantations. The presence and distribution of several aminopeptidases including A-LAP and ERAP1 have been shown to be related with cell proliferation and differentiation of human endometrium (Shibata *et al*. 2004). In our results, significant changes in expression level of ERAP1 were detected in Ishikawa cells treated with 10^−7^ M E_2_. Future studies will be directed to delineate the hormone-induced physiological changes in kallikrein–kinin system and aminopeptidases in various phases of the endometrium. Additionally, a key event in the eukaryotic gene regulation is the post-translational acetylation of nucleosomal histones upon decidualization (Sakai *et al*. 2003). Consistent with the most recent data (Piras *et al*. 2017), we detected increased expression levels of histones (H_1_–H_4_) in cells treated with 10^−7^ M E_2_. In women undergoing IVF treatment, metabolomic analysis of follicular fluid revealed a decrease in levels of choline, glycerophosphocholine and phosphocholine in patients whose fertilized oocyte failed to cleave to an embryo (Wallace *et al*. 2012). In contrast, 10^−7^ M E_2_ treatment significantly downregulated expression level of choline in human endometrial epithelial cells *in vitro*. IPA program is applied to determine predicted upstream regulators, signaling pathways or group of proteins identified by iTRAQ. In the present study, IPA generated top connectivity network of 45 differentially expressed proteins. Proteins including highlighted C_3_, SERPINs, PLG, KNG1 and number of other proteins in the confirmed network might change the metabolic status of the endometrium toward a receptive stage. Downstream functional enrichment analysis of cellular function and maintenance showed that most proteins in this network were upregulated in the human endometrial epithelial cells treated with 10^−7^ M E_2_, and these upregulated proteins were related to cellular homeostasis. After analyzing the association of the differentially expressed proteins with cellular functions and maintenance, we found that 14 proteins could be categorized as being involved in cellular homeostasis and 9 proteins could be categorized as being involved in ion homeostasis of cells. Differentially expressed proteins detected in present study also mediated signaling pathways such as the LXR/RXR activation pathway and the acute-phase response signaling pathway involved in lipid metabolism and inflammation (Mouzat *et al*. 2009, Birse *et al*. 2013) respectively. In mouse endometrium and myometrium, two isoforms of liver X receptors (LXRa and LXRb) are expressed, suggesting the existence of a molecular link between cholesterol levels, LXRs and deregulation of ovulation, particularly in ovarian hyperstimulation syndrome (OHSS) (Mouzat *et al*. 2009). *In vivo* study showed that Lxr-deficient (−/−) mice offered some signs of infertility (Steffensen *et al*. 2006). RXR was found to be constitutively expressed throughout the gestation (Plösch *et al*. 2010). Furthermore, clinical studies are needed to explain their role in OHSS patients. Our upstream analysis listed several upstream regulators identified in Ishikawa cells treated with 10^−7^ M E_2_. However, extensive investigations are required to elucidate their biological significance by validating target genes relevant to implantation and pregnancy.

## Conclusions

In summary, the systematic analyses provide the basis for understanding the estrogen-dependent changes in protein profiles of endometrial compartments with some distinct proteins relevant to uterine receptivity and suggest that optimized dosage fashion of estrogen regimen may maintain high pregnancy rates and probable target endometrium at the endometrial–embryonic interface in high responders. Moreover, to determine sufficient management and to validate minimal ovarian hyperstimulation along with late embryo transfer, further molecular and clinical researches are needed to clarify the underlying mechanisms of different effects of estrogen at different doses on embryo implantation in COH patients.

## Declaration of interest

The authors declare that there is no conflict of interest that could be perceived as prejudicing the impartiality of the research reported.

## Funding

This study was supported by the National Basic Research Program of China (No. 2012CB944900 to H F H), the Natural Science Foundation of China (81450038, 31171444 to H F H; 81300458 to T T W and 81270708 to J Z S), NSFC-CIHR Joint Health Research Programs (81361128007 to J Z S) and a Canadian Institutes for Health Research China-Canada Joint Health Research Initiative grant (CCI-132570 to P C K L).

## Authors’ contribution statement

K U, H F H and J Z S designed the experiment. K U, T U R, H T P, X Y D, L Y J, J L and Y C performed the experiments. K U, M X G, Z H K, J R, X H L, X X Q and T T W acquired and analyzed the data. K U, T U R, H F H and J Z S wrote the manuscript.
